# Tiny Regulators of Massive Tissue: MicroRNAs in Skeletal Muscle Development, Myopathies, and Cancer Cachexia

**DOI:** 10.3389/fonc.2020.598964

**Published:** 2020-11-23

**Authors:** Gurinder Bir Singh, Douglas B Cowan, Da-Zhi Wang

**Affiliations:** Department of Cardiology, Boston Children’s Hospital, Harvard Medical School, Boston, MA, United States

**Keywords:** microRNA, skeletal muscle, cachexia, Duchenne muscular dystrophy, sarcopenia

## Abstract

Skeletal muscles are the largest tissues in our body and the physiological function of muscle is essential to every aspect of life. The regulation of development, homeostasis, and metabolism is critical for the proper functioning of skeletal muscle. Consequently, understanding the processes involved in the regulation of myogenesis is of great interest. Non-coding RNAs especially microRNAs (miRNAs) are important regulators of gene expression and function. MiRNAs are small (~22 nucleotides long) noncoding RNAs known to negatively regulate target gene expression post-transcriptionally and are abundantly expressed in skeletal muscle. Gain- and loss-of function studies have revealed important roles of this class of small molecules in muscle biology and disease. In this review, we summarize the latest research that explores the role of miRNAs in skeletal muscle development, gene expression, and function as well as in muscle disorders like sarcopenia and Duchenne muscular dystrophy (DMD). Continuing with the theme of the current review series, we also briefly discuss the role of miRNAs in cancer cachexia.

## Introduction

Skeletal muscle consists of approximately 42 and 36% of the average body weight of male and female humans, respectively. It is a highly plastic tissue with an ability to contract and stretch. Apart from playing an important role in supporting movement and respiration, skeletal muscle also acts as a reservoir for proteins, generating body heat, and protecting other internal tissues and organs (1, 2). As a result, maintaining the proper function of skeletal muscle is indispensable and understanding the factors that regulate muscle mass is essential.

Altered expression of microRNAs has been shown to be involved in skeletal muscle homeostasis in health and disease (3–6). MicroRNAs (miRNAs) are small (~22 nucleotides long), ubiquitously expressed non-coding RNA sequences involved in regulating gene expression at the post-transcriptional level. MiRNAs are known to regulate gene expression by binding to the 3’ untranslated region (3’UTR) of targeting mRNAs resulting in either their degradation or inhibition of protein translation. A few reports have also indicated the possible binding of miRNAs to the 5’UTR or coding region of target mRNAs. MiRNAs are transcribed by RNA polymerase II or III as a long primary miRNA (pri-miRNA) from different regions of the genome, including non-coding regions (intronic or intergenic) and protein coding regions (exons) as an individual miRNA (monocistronic) or cluster (poly-cistronic) (7).

Pri-miRNAs are processed in the nucleus by the RNase III endonuclease Drosha and its cofactor Di George syndrome critical region 8 gene (DGCR8) into short ~70 nucleotide precursor miRNAs (pre-miRNAs) (8). Pre-miRNAs are exported *via* exportin-5 into the cytoplasm, where it is cleaved by the RNase III enzyme Dicer, yielding approximately 22 nucleotide long imperfect miRNA duplexes (ds-miRNAs) (9). Helicase unwinding of these ds-miRNAs results in a miRNA (*i.e.* the leading or mature strand) and a miRNA* (*i.e.* the passenger strand) (10). The mature strand of the miRNA/miRNA* duplex is incorporated into the RNA-induced silencing complex (RISC) containing Dicer and AGO2 (argonaute RISC catalytic component 2) along with RNA binding proteins, while the miRNA* strand is mostly degraded. The RISC is guided by the mature miRNA to the 3’UTR of target mRNAs, resulting in either degradation or translational inhibition (11, 12).

While the role of miRNAs is well documented in many biological events, including cell death, differentiation, proliferation, and cell growth (13, 14), miRNAs have also emerged as important regulators of various pathways involved in skeletal muscle development and function. The present review summarizes the latest research exploring the role of miRNAs in skeletal muscle myogenesis and describes their role in muscle disorders like sarcopenia and Duchenne muscular dystrophy (DMD). In keeping with the theme of the current review series, we have also included a section describing the role of miRNAs in cancer cachexia.

## MiRNAs and Myogenesis

The process of generating skeletal muscle is called myogenesis. The development and growth of muscle is regulated by important muscle-specific transcription factors known as myogenic regulatory factors (MRFs). Expression of MRFs are limited to the muscle lineage leading to the formation of muscle fibers following activation of downstream signaling pathways. During embryogenesis, the paired box transcription factors Pax3 and Pax7 regulate early MRFs. MRFs include transcription factors of the MyoD family, such as myogenin, Myf5, MyoD, MRF4 (also called Myf6), as well as the MEF2 family and the serum response factor (SRF) (15–17).

Many studies have highlighted the role of muscle-specific microRNAs in myogenesis with an emphasis on their role in the signaling pathways involved in muscle cell (myocyte) proliferation and differentiation by acting either synergistically or antagonistically (18, 19). For example, a report by O’Rourke et al. demonstrated the importance of miRNAs in skeletal muscle development. In this study, the authors generated mice with a skeletal muscle-specific deletion of *Dicer*, which encodes an important enzyme involved in miRNA biogenesis. *Dicer* knock-out mice showed significantly decreased expression of many muscle-specific miRNAs resulting in muscle hypoplasia and lethality—highlighting the importance of miRNAs in muscle development (20).

## MyomiRs and Skeletal Muscle

There are two groups of miRNAs involved in the regulation and development of skeletal muscle. The first group is exclusively expressed in muscle, called myomiRs, while the second group is expressed in both muscle and non-muscle cells, called non-myomiRs. Much of our understanding of miRNAs in skeletal muscle initially focused on myomiRs, which are expressed or enriched in the heart and skeletal muscles. These myomiRs included eight miRNAs; miR-1, miR-133a/b, miR-208b, miR-486, and miR-499 (expressed in the myocardium and skeletal muscle), while miR-208a was expressed exclusively in the myocardium and miR-206 was restricted to skeletal muscle (21) (**Table 1**).

**Table 1:** MyomiRs Involved in Skeletal Muscle Myogenesis.

MyomiR	Target genes	Biological role	References
miR-1	*Pax3*, *Pax7*, *HDAC4*, *Cx43*, *YY1*, *CNN3*, *SFRP1*, *Notch3*	Myoblast differentiation and regeneration	(22–26)
mir-133	*SRF*, *nPTB*, *IGF*‐*1R*, *UCP2*, *Foxl2*, *FGFR1*, *PP2AC*	Myoblast proliferation	(23, 25, 27–29)
miR-206	*DNA pola1*, *Pax3*, *Pax7*, *Cx43*, *Utrn*, *Fstl1*, *HDAC4*, *nPTB*	Myoblast differentiation and regeneration	(24, 25, 30, 31)
miR-208b	*Sox6*	Specification of muscle fibers	(32)
miR-486	*PTEN*, *FoxO1a*, *Pax7*	Myoblast differentiation	(33)
miR-499	*Sox6*	Specification of muscle fibers	(32)

Early studies in skeletal muscle C2C12 myoblasts highlighted the importance of myomiRs in the differentiation and development of skeletal muscle (22). The myogenic regulatory factors SRF, MyoD, MyoG, MEF2, and myogenin directly regulate expression of myomiRs (34–36). The miR-1/206 family is comprised of miR-1-1, miR-1-2, and miR-206. MiR-1 and miR-6 share identical seed sequences and differ by only four nucleotides outside the seed region (23). The importance of miR-1 and miR-206 in myoblast differentiation and muscle development has been well described. For example, Kim et al. showed an increase in the expression of miR-1 and miR-206 promotes the differentiation of satellite cells (30).

MyomiRs promote muscle cell differentiation and development by targeting repressors of skeletal muscle cell differentiation. MiR-1 promotes myogenesis by targeting histone deacetylase 4 (HDAC4) and a transcriptional repressor of the muscle-specific transcription factor, MEF2; thereby, promoting expression of MEF2, which increases the expression of miR-1 and accelerates the differentiation of muscle fibers (23, 24). Similarly, miR-206 was reported to enhance the differentiation of myoblasts by repressing DNA polymerase alpha (*Polα1*), the gap junction protein connexin43, follistatin-like 1 protein (*Fstl1*), and utrophin (*Utrn*). Polα1 is involved in DNA synthesis, and its inhibition reduces DNA synthesis at the initial differentiation stages and promotes myocyte differentiation by restricting cell proliferation. By targeting connexin43 (*Cx43*), miR-206 acts to reduce intercellular communication between muscle fibers (30, 31, 37, 38). Utrn and Fstl1 are also involved in myoblast proliferation and an important target of miR-206 (31). Recently, Ma et al. reported *nPTB* is another key target for miR-206 and this interaction affects differentiation (25). Therefore, miR-1 and miR-206 regulate many common targets like *Pax3*, *Pax7*, *HDAC4*, *Cx43*, and *Notch3* (23–25, 39), while *YY1*, *CNN3* (calponin 3), *SFRP1* are the exclusive targets of miR-1 (22, 26).

Another important member of the myomiR family is miR-133, which includes miR-133a-1, miR-133a-2, and miR-133b (40). Their expression is up-regulated during myogenesis, similar to miR-1 (23, 41). However, unlike miR-1, which promotes myogenic differentiation, miR-133 is primarily reported to be involved in muscle cell proliferation and inhibition of myocyte differentiation. MiR-133 promotes myocyte proliferation by targeting serum response factor (*SRF*) and decreasing its expression (23). In particular, many reports have recently examined the role of miR-133 in myocyte differentiation.

Neuronal polypyrimidine tract‐binding protein (nPTB) and uncoupling protein 2 (UCP 2) are negative regulators of myocyte differentiation. MiR-133 is reported to promote differentiation by targeting *nPTB* (25) and *UCP-2* (27). Moreover, the inhibition of the forkhead box transcription factor 2 (*Fox 2*) by miR-133 can also promote differentiation (42). Fibroblast growth factor receptor 1 (FGFR1) and protein phosphatase 2A (PP2AC) expression is important for regulating the ERK1/2 signal transduction pathway. Inhibition of ERK1/2 signaling plays an important role in myocyte differentiation and miR-133 promotes muscle cell differentiation by targeting *FGFR1* and *PP2AC* (28).

MiR-486 is another muscle enriched miRNA and it is significantly increased during muscle cell differentiation. In both satellite cells and C2C12 cells, miR-486 can promote differentiation by targeting *Pax7* (33). MiR-486 also targets regulators of PI3K/AKT signaling, *PTEN* (phosphatase and tensin homolog), and *FoxO1a* (forkhead box protein O1a). PI3K/AKT signaling is involved in repression of skeletal myogenesis and the inhibition of *PTEN* and *FoxO1a* by miR-486 promotes myogenesis (43).

At the same time, miR-208 and miR-499 are involved in the differentiation of myocytes into type I slow twitch and type II fast twitch muscle myofibrils. MiR-208a is expressed exclusively in heart, whereas miR-208b and miR-499 are expressed in both the heart and skeletal muscle. They are expressed from intronic sequences embedded within the myosin heavy chain genes *Myh6*, *Myh77*, and *Myh7B*, respectively (32). These miRNAs broadly participate in the regulation of muscle gene expression, myofiber specification, and muscle function as double knockout mice for miR-208 and miR-499 showed increased expression of SRY-box containing gene 6 (*Sox6*), resulting in decreased expression of Myh7 and loss of slow type I slow twitch myofibers (32).

In addition to myomiRs many more miRs are expressed in skeletal muscle and they play a significant role in the regulation of myogenesis. These non-myomiRs (meaning their expression is not restricted to muscle cells), are known to control many processes involved in muscle, like muscle satellite cell (MSC) differentiation and proliferation, myoblast differentiation and proliferation, and myofibril formation. Consequently, the role of non-myomiRs in skeletal muscle regulation is becoming well-documented (19, 44, 45). **Table 2** highlights the role of many of these miRs in skeletal muscle.

**Table 2:** MiRNAs that regulate muscle development.

	Non -myomiRs	Target gene	Function	Reference
**Muscle Satellite Cells (MSCs)** **regulation**	miR-9-5p	*IGF2BP3*	Proliferation and differentiation of MSCs	(46)
	miR-27	*Pax 3*	Increases differentiation and proliferation of MSCs.	(27, 29)
	miR-31	*Myf5*	Delays activation of the myogenic program and subsequent differentiation.	(47)
	miR-106b	*Myf5*	Delays activation of the myogenic program and subsequent differentiation.	(48)
	miR-195/497	*Cyclin E1*, *cyclin D2*, *Cdc25*	Maintains the quiescent state of MSCs.	(49, 50)
	miR-431	*Pax7*	Promotes myogenic differentiation.	(51)
	miR-378	*POLA2*	Bovine skeletal muscle development	(52)
**Myoblast proliferation and differentiation**	miR-16-5p	*SESN1*	Represses myoblast differentiation.	(53)
	miR-17-92	*ENH1*	Promotes myoblast proliferation	(54)
	miR-20a/b	*E2F1*, *pri-miR-17-92* and *pri-miR-106a-363*	Represses myoblast proliferation.	(55)
	miR-22	*TGF‐βR1*	Promotes myocyte differentiation.	(56)
	miR-23a	*Myh 1, 2* and *4*	Inhibits myoblast differentiation.	(57)
	miR-24	*SMAD2*	Regulates myogenic differentiation.	(58, 59)
	miR-26	*SMAD1*, *SMAD4* and *Ezh2*	Promotes differentiation of myoblasts.	(60)
	miR-29	*YY1*, *Rybp*	Promotes myoblast differentiation.	(61, 62)
	miR-34c	*YY1*	Represses myoblast proliferation.	(63)
	miR-98	*E2F5*	Represses myoblast differentiation.	(64)
	miR-221	*p27*	Modulate differentiation and maturation of MSC	(65)
	miR-222	*p27*	Modulate differentiation and maturation of MSC	(65)
	miR-128	*IRS1*, *Pik3r1*, *Insr*	Promotes myoblast proliferation.	(66)
	miR-139	*Wnt1*	Promotes myoblast proliferation and repress differentiation.	(67, 68)
	miR‐ 145a‐ 5p		Promotes myoblast differentiation.	(69)
	miR-148a	*ROCK1*	Promotes myoblast differentiation	(70)
	miR-151-3p	*ATP2a2*	Promotes myoblast proliferation	(50)
	miR-155	*Mef2a*	Inhibits myoblast differentiation	(71)
	miR-181	*Hox-A11*	Enhances muscle differentiation.	(72)
	miR-186	*Myog 4*	Inhibits myoblast differentiation	(73)
	miR-195/497	*IGF‐1R*	Promotes myoblast Proliferation	(50)
	miR-199-3p	*IGF-1*, *mTOR*, *RPS6KA6*	Represses myoblast differentiation.	(74)
	miR-199-5p	*FZD4, JAG1*, *Wnt2*	Increases proliferation of myoblast.	(75)
	miR-203	*c‐JUN*, *MEF2C*	Represses myocyte proliferation and induces cell cycle arrest	(76, 77)
	miR-223	*IGF2*, *ZEB1*	Inhibits myoblast proliferation.	(78)
	miR-322/424	*Cdc25*	Cell cycle regulator promotes cell cycle quiescence and differentiation	(79)
	miR-351	*lactamase β*	Promotes myoblast proliferation	(69)
	miR-374	*Myf6*	Represses myoblast differentiation.	(25)
	miR-378a-3p	*HDAC4*	Promotes differentiation of Myoblasts.	(80)
	miR-431	*SMAD4*	Promotes myoblasts differentiation.	(21, 81)
	miR-503	*Cdc25*	Cell cycle regulator promotes cell cycle quiescence and differentiation	(79)

## Role of miRNAs in Satellite Cells and Skeletal Muscle Regeneration

MiRNAs have been shown to play a vital role in the regulation of satellite cells and skeletal muscle myogenesis by targeting various muscle-specific transcription factors and important signaling pathways. Previous studies have highlighted the role of miRNAs in skeletal muscle cell regulation, suggesting a vital role for these miRNAs in satellite cell-mediated muscle development. The paired-box transcription factors Pax 3 and Pax 7 are crucial for satellite cell proliferation and differentiation. MiR-27b is highly expressed in activated satellite cells and it directly targets *Pax3* mRNA and, consequently, promotes satellite cell differentiation while inhibiting proliferation (47). MiR-431 is another miRNA shown to suppress the proliferation of satellite cells and enhance differentiation by targeting *Pax-7* mRNA. Interestingly, in miR-431 overexpressing transgenic mice, there was significant improvement in muscle regeneration compared to control mice.

Other important miRNAs include miR-195 and miR-497, which are members of the miR-15 family, which maintains the quiescent state of satellite cells. MiR-195 and miR-497 share the same seed sequences and target the cell cycle activators *Ccnd2*, *Cdc25*, *cyclin D1*, and *cyclin D2* (49, 50). MiR-31 is also reported to maintain the quiescent state of satellite cells as it targets *Myf5*, an important myogenic regulator needed for the differentiation of satellite cells toward the myogenic lineage, which delays differentiation (47). Similarly, a report by Velasco et al. showed targeting of *Myf5* mRNA by another miRNA (miR-106b) delays activation of the myogenic program and, subsequently, differentiation (48).

## Role of miRNAs in Myoblast Differentiation and Proliferation by Targeting MRFS

**Figure 1:**
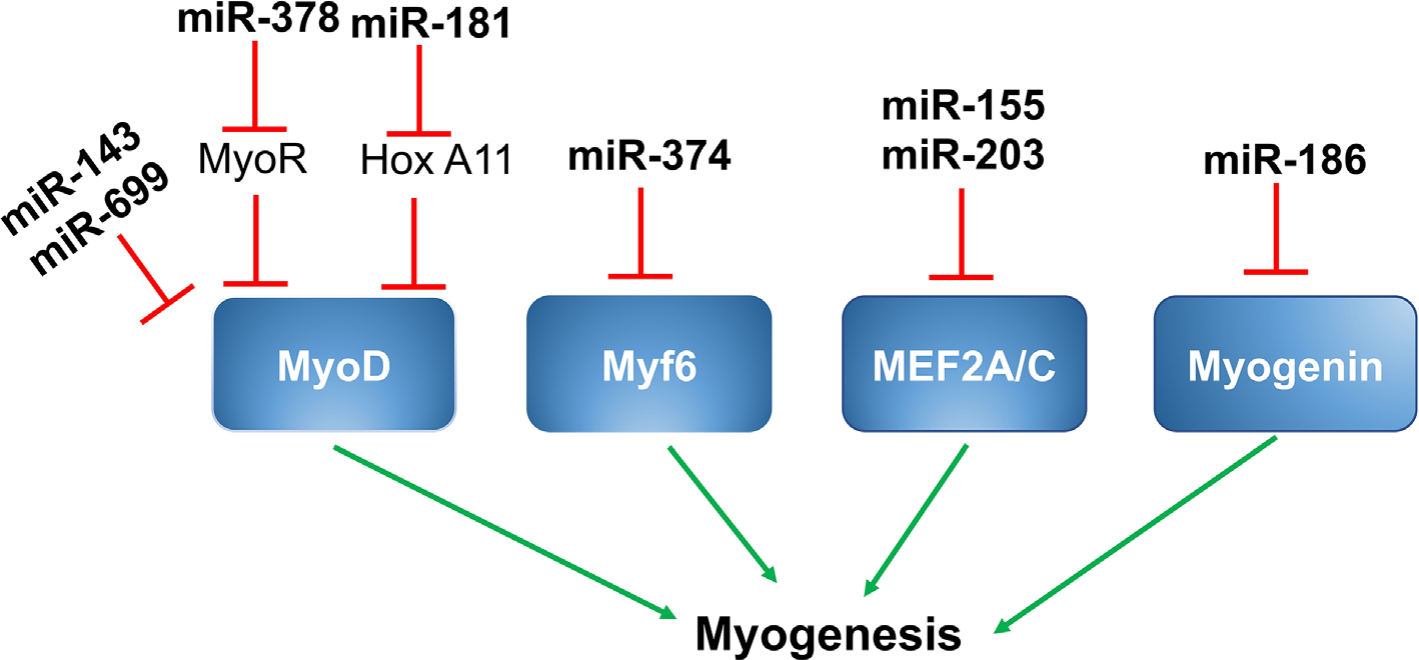
MiRNAs and myogenic regulatory factors. MiR-143 and miR-699 targets *MyoD* and suppress myogenesis. MiR-378 is up-regulated by MyoD, which targets MyoR and further upregulates MyoD through positive feedback. MiR-181 targets *Hox-A11*, leading to increased expression of MyoD and myogenin. MiR-376 targets *Myf6* and suppresses myogenesis. MiR-155 and miR-203 suppresses myogenesis by targeting *MEF2A* and *MEF2C* respectively. MiR-186 targets myogenesis and controls myogenesis.

Given their critical roles in the regulation of muscle gene expression as well as the myogenic program and muscle function, it is not surprising that myogenic regulatory factors (MRFs), like MyoD, Myf5, Myf6, myogenin, and members of the myocyte enhancer factor 2 (MEF2) family of transcription factors, including MEF2A, MEF2B, MEF2C, and MEF2D, are direct targets of muscle-specific miRNAs, resulting in alteration of muscle gene expression in skeletal muscle (**Figure 1**).

## MiR‐143/MyoD

A study by Chen et al. showed miR‐143 was a direct target of *MyoD* in the skeletal muscles of *Siniperca chuatsi*, a type of Asian Perch. Expression of miR-143 was inversely proportional to the expression of *MyoD* in fast and slow twitch muscles. As suppression of miR‐143 results in a substantial increase in *MyoD* and fast *MyhC* gene expression *in vivo*, miR143 likely has an important role in the differentiation of different muscle fiber types in vertebrates (82).

## MiR-378/MyoR/MyoD

Expression of miR-378 is induced during the differentiation of C2C12 cells in culture. MiR-378 promotes skeletal muscle cell differentiation by decreasing the expression of negative regulators. MiR-378 is encoded within an intron of the peroxisome proliferator-activated receptor gamma coactivator 1-beta (*PPARγ-c1β*) that encodes for PGC-1β. Expression of PGC-1β/miR-378 is regulated by MyoD, which binds to the regulatory sequences upstream of PGC-1β/miR378. Mir-378, in turn, binds to the MyoD transcription repressor MyoR and, thereby, promotes MyoD expression in a positive feedback loop (39).

## MiR-181/HoX-A11/MyoD

miR-181 is another microRNA that promotes skeletal muscle differentiation by decreasing the expression of negative regulators. Expression of miR-181 is elevated in differentiating myoblasts and is less expressed in terminally differentiated muscle cells. In skeletal muscle cells, increased expression of MyoD suppresses cell regeneration and promotes terminal differentiation. The target gene for miR-181 is homeobox protein called *Hox-A11*, which is a well-known muscle cell differentiation repressor because it suppresses the expression of MyoD. MiR-181 targets *Hox-A11* and, ultimately, leads to increased expression of MyoD; thereby, promoting muscle cell terminal differentiation. In C2C12 myoblasts, inhibition of miR-181 suppresses myoblast differentiation by reducing MyoD expression (72, 83).

## MiR-669a, miR-699q/MyoD

Two members of miR669 family, miR669a and miR669q, are involved in cardiac skeletal muscle differentiation. Both miR669a and miR669q directly target *MyoD* and decrease the expression of MyoD and muscle cell differentiation (21).

## MiR-374/Myf6

MiR-374 is a miRNA that regulates myoblast differentiation by targeting another myogenic regulatory factor, Myf6. Myf6 regulates the expression of genes involved in myoblast terminal differentiation. MiR-374 directly binds to the 3’ UTR region of *Myf6* and decreases its expression. In mouse skeletal muscle and C2C12 myoblasts, miR-374 regulates differentiation. As shown by Ma *et al.*, overexpression of miR-374 suppresses myoblast differentiation, while its inhibition using by 2’-O-methyl antisense oligonucleotides promotes C2C12 myoblast differentiation (25).

## MiR-186/Myogenin

Myogenin is another important MRF involved in the regulation of myoblast differentiation. It plays a critical role in the regulation of terminal differentiation of the muscle cells. MiR-186 directly binds to the 3’ UTR region of *myogenin* and decreases its expression at both the mRNA and protein levels. MiR-186 resides between exons 8 and 9 of the ZRANB2 gene and these miRNAs are transcribed in parallel with the host gene. Overexpression and inhibition studies in C2C12 myoblasts as well as in primary muscle cells showed that inhibiting myogenin expression prevented differentiation (73).

## MiR-155/MEF2A

MEF2A is a member of the MEF2 (myocyte enhancer factor 2) family of transcription factors and plays a positive role in muscle cell differentiation. MiR-155 directly binds to the 3’ UTR region of *MEF2A* and decreases its expression at both the mRNA and protein levels. As demonstrated by Seok et al., overexpression of miR-155 significantly down-regulates the expression of MEF2A and, thus, decreases myoblast differentiation. Also, when MEF2A is reintroduced into miR-155 overexpressing myoblasts, it partially promotes differentiation (71).

## MiR-203/MEF2C

MiRNA-203 is a miRNA that plays a regulatory role in myoblast proliferation and differentiation. During differentiation of myoblasts into myotubes, miR-203 is significantly down-regulated. As shown by Lu et al., miR-203 overexpression suppresses myoblast proliferation and differentiation, and its inhibition promotes proliferation and differentiation. *MEF2C* and *c-Jun* (a transcriptional activator of the AP‐1 family) are the direct targets of miR-203. C-Jun is known to promote muscle cell proliferation and MEF2C is well-known to promote differentiation. Therefore, miR-203 can regulate both proliferation and differentiation during muscle development (76, 77).

## MiR-23a/Myh

During myogenic differentiation, expression of the myosin heavy chain (Myh) is regulated by muscle related transcription factor MEF2C (84). MiR-23a suppresses myoblast differentiation in C2C12 myoblasts. *Myh* gene isoforms *Myh* 1, 2, and 4 are the direct targets of miR-23a. Overexpression and inhibition studies in C2C12 myoblasts further confirmed that miR-23a decreases the expression of Myh isoforms and inhibits myoblast differentiation (57). A study by Mercatelli et al. showed that miR-23a and miR-23b target thioredoxin reductase 1 (*Trxr1*). Overexpression of Trxr1 delays differentiation by negatively modulating the expression of myogenin and Myh genes (85).

## MiRNAs in Myoblast Differentiation and Proliferation by Signal Regulation

Apart from targeting MRFs, miRNAs also regulate myoblast proliferation and differentiation by targeting genes encoding key components of various signaling pathways. In the following section, we will discuss various signaling pathways like IDI (inhibitor of differentiation 1) signaling, TGF-β/SMAD/BMP signaling, insulin signaling, and NF-κB-YY1 signaling in addition to the role of associated miRNAs in the regulation of skeletal muscle development.

## MiR-17-92/E2F1/ID1 Signaling

The miR‐17-92 cluster consists of miR-17, miR-20a, and miR-92a, and these miRNAs promote myoblast proliferation and suppress differentiation along with tube formation. Transcription of the miR-17-92 cluster is regulated by E2F1. Actin-associated protein enigma homolog 1 (*ENH1*) and transcription factor *E2F3b* (E2F transcription factor 3b) are the direct targets of microRNAs from the miR-17-92 cluster. MiR-17-92 suppresses ENH1 by directly targeting its 3’UTR and results in increased expression of ID1 in the nucleus. ID1 binds to MRF and E protein and subsequently suppresses myogenic differentiation. Another target of the miR-17-92 cluster *is E2F3b*. Inhibition of E2F3b further suppresses myotube formation (54, 86).

## MiR-98/E2F5-ID1 Signaling

**Figure 2:**
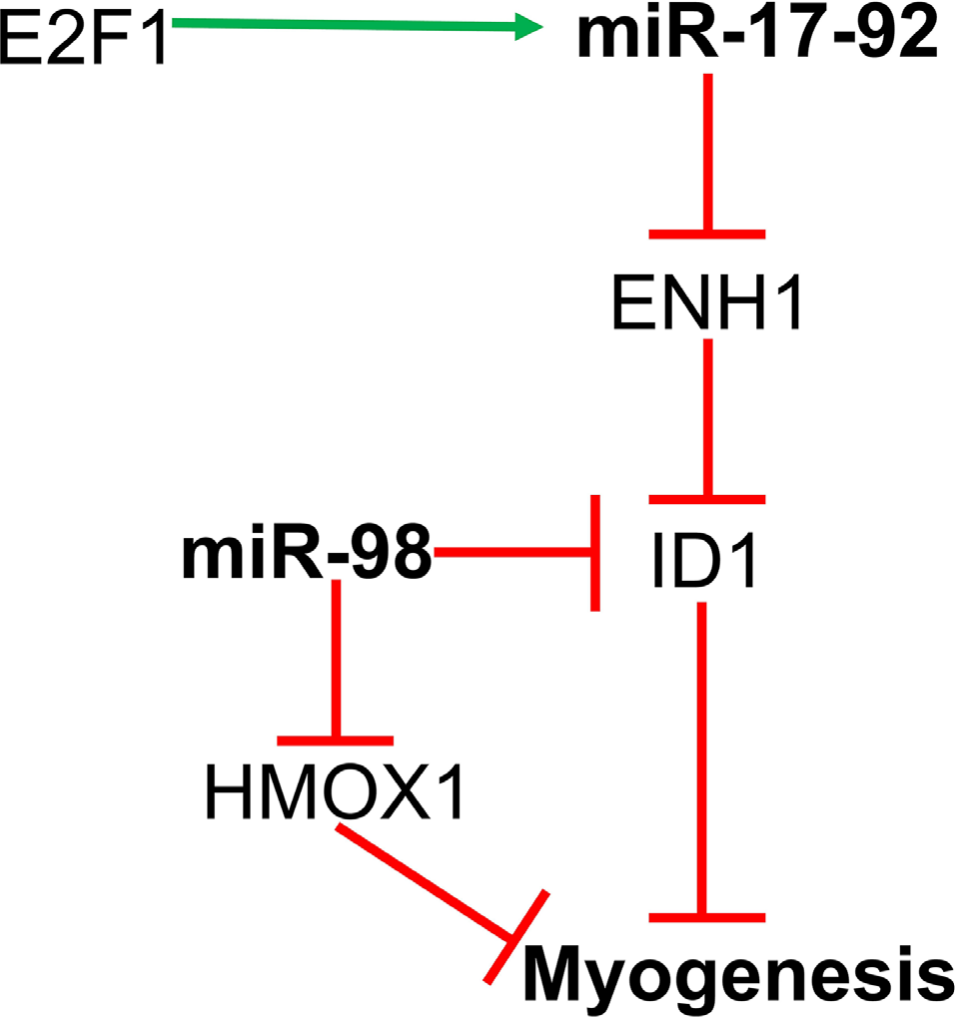
MiR-17-92 and miR-98 regulates myoblast proliferation and differentiation by targeting the ENH1/Id1 signaling axis.

MiR-98 is involved in negatively regulating myogenic differentiation of C2C12 myoblasts. E2F5 is a transcriptional repressor and miR-98 directly targets the 3’ UTR region of *E2F5*. Kropp et al. showed that simultaneous inhibition of both miR-98 as well as E2F5 in C2C12 myoblasts restores differentiation. Also, E2F5 itself binds to the promoters of ID1 and HO-1 (heme oxygenase 1), both of which are inhibitors of terminal muscle cell differentiation resulting from their decreased expression (64). Therefore, miR-98 regulates myoblast differentiation *via* E2F5, which itself regulates ID1 and HO-1 (**Figure 2**).

## MiR‐22, miR-24, miR-26, miR-146, miR-431, and miR-375 in TGF-β/SMAD/BMP Signaling

Expression of miR-22 is up-regulated during C2C12 myoblast differentiation. MiR-22 regulates muscle differentiation by targeting transforming growth factor β receptor 1 (*TGFβ-R1*). TGFβ-R1 is an important receptor of the TGF-β/SMADSMAD signaling pathway. Overexpression of miR-22 in C2C12 down-regulates expression of TGFβ-R1 and promotes myoblast differentiation into myotubes; whereas, inhibition of miR-22 suppresses myoblast differentiation and promotes proliferation. Transforming Growth Factor β-1 (TGF-β1) could also regulate the expression of miR-22 in C2C12 cells as treatment of C2C12 cells with TGF-β1 decreases the level of miR-22, highlighting a significant role of TGF-β1/SMAD3 signaling in myogenic differentiation (56).

MiR-24 is another miRNA involved in myogenic differentiation. Overexpression of miR-24 in C2C12 myoblasts significantly promotes differentiation and myotube formation, and differentiation is suppressed upon treatment with TGF-β1. Recently, a report by Sun et al., suggested that miR-24 may regulate TGF-β/SMAD signaling by targeting *SMAD2* (58). MiR-26 is another non-myomiR that regulates myoblast differentiation by targeting TGF-β/SMAD signaling. MiR-26 also targets the *BNP* (bone morphogenetic protein) of the SMAD pathway, which regulates myoblast differentiation. TGF-β/BMP signaling helps to maintain a pool of muscle stem cells by preventing myogenic differentiation.

*SMAD1* and *SMAD4* are direct targets of miR-26 and overexpression of miR-26 promotes differentiation of myoblasts, while miR-26 inhibition suppresses myoblast differentiation. Thus, miR-26 regulates myoblast differentiation through the TGF-β/BNP signaling pathway (60). Additionally, miR-26 targets the histone methyltransferase *Ezh2* (enhancer of zeste homologue 2) and enhances differentiation of C2C12 myoblasts (87). MiR-146b is another miRNA involved in the regulation of myoblast differentiation (88). A study by Khanna et al. showed that *SMAD4*, *Notch1*, and *Hmga2* are direct targets of miR-146b.

As discussed above, SMAD4 is involved in the TGFβ/BMP pathway and regulates myoblast proliferation and differentiation (89). Furthermore, Notch signaling is known to control satellite cell quiescence, and inhibition of Notch by miR-146 promotes myoblast differentiation (90). Similarly, inhibition of *Hmga2* by miR-146b also promotes myoblast differentiation as Hmga2 is a key regulator of satellite cell activation and proliferation (91). Hence, miR-146b enhances myogenic differentiation through multiple targets. MiR-431 is another microRNA targeting *SMAD4*, which, in turn, regulates TGFβ signaling. MiR-431 is shown to be present at lower levels in the skeletal muscle of aged mice compared to young mice. Also, increased expression of miR-431 in an *in vivo* model of muscle regeneration significantly enhances regeneration and reduces SMAD4 levels; thereby, increasing myogenic differentiation (81).

**Figure 3:**
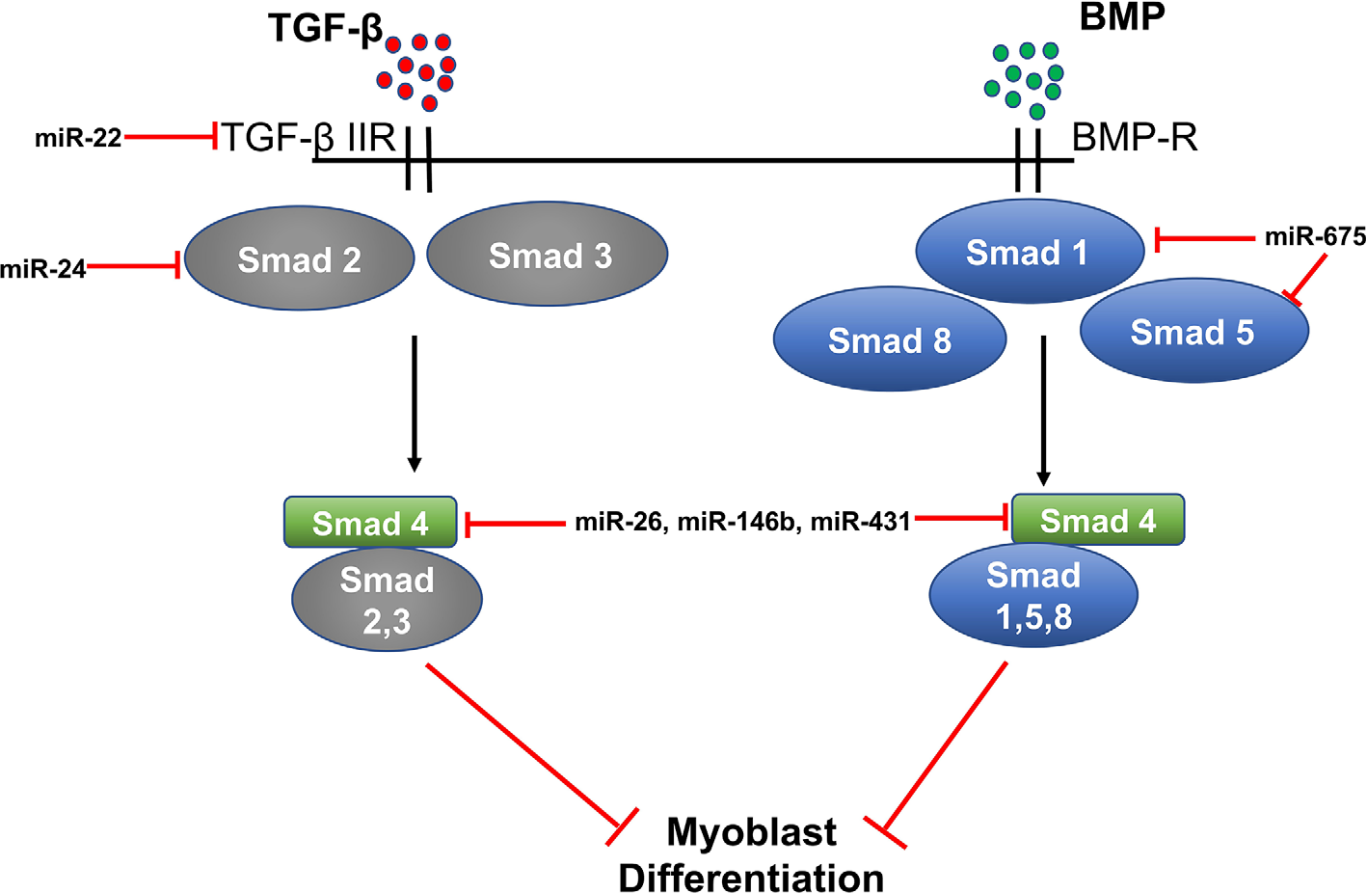
MiRNA regulation of TGF-β/SMAD/BMP signaling in myogenesis.

Two miRNAs, miR-675-3p and miR675-5p, are induced during skeletal muscle differentiation. These miRNAs are encoded by exon one of the long non-coding RNA H19. H19 is known to regulate myoblast differentiation and regeneration because its knockdown in myoblasts decreases their differentiation and regeneration potential. H-19-deficient mice show decreased muscle regeneration and differentiation, which is rescued by the re-introduction of miR-675-3p and miR-675-5p. *SMAD1* and *SMAD5* transcription factors and the DNA replication initiation factor *Cdc6* are direct targets of miR-675-3p and miR-675-5p and these miRNAs decrease expression of SMAD transcription factors involved in the anti-differentiation BMP signaling pathway (**Figure 3**) (92).

## MiR-128 and Insulin Signaling

**Figure 4:**
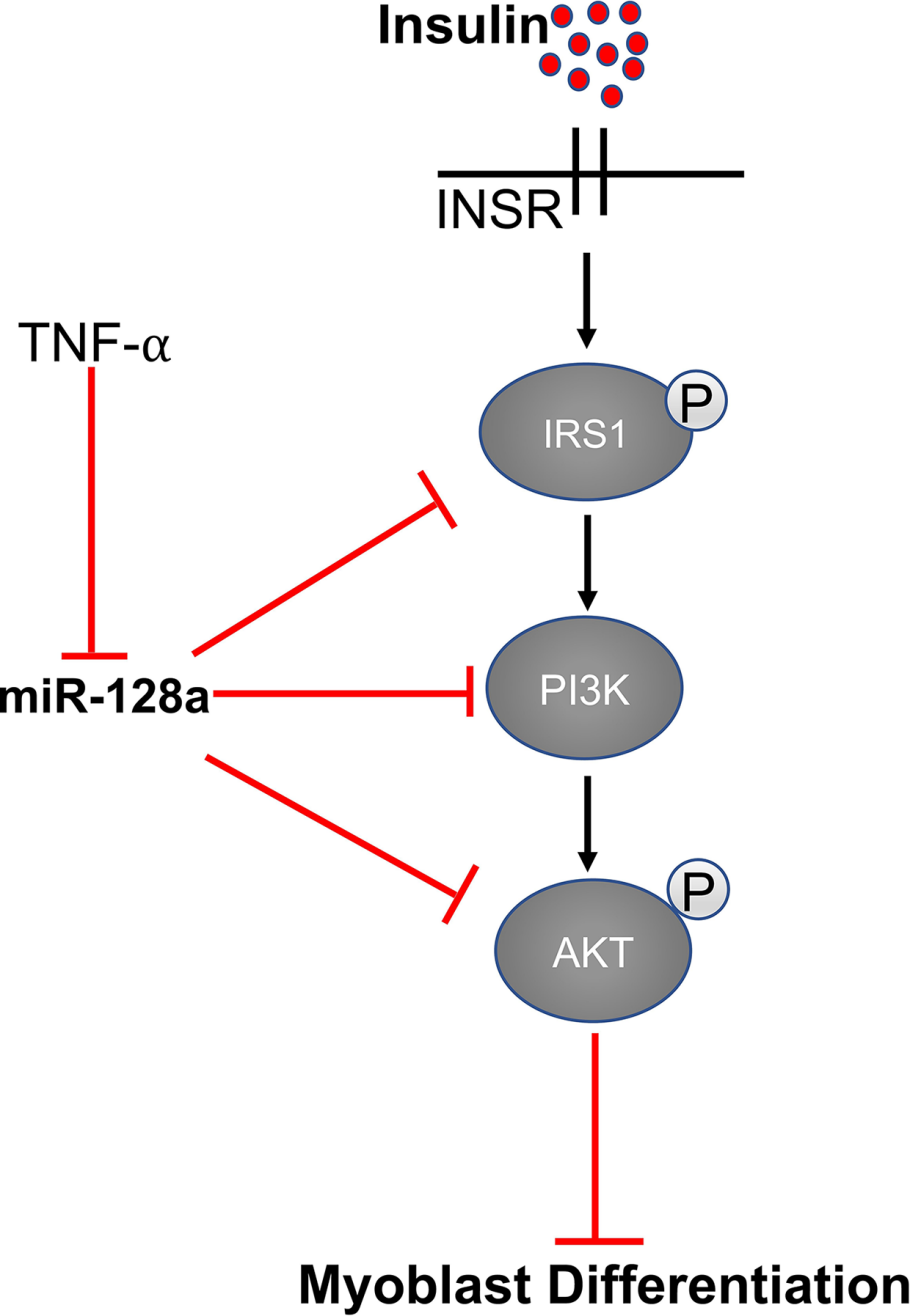
MiR-128 regulates the expression of IRS1, AKT, and PI3K.

Expression of miR-128 is significantly up-regulated during myoblast differentiation in skeletal muscle. IR (insulin receptor), IRS-1 (insulin receptor substrate 1), and PI3K-R1* *(phosphatidylinositol 3-kinases receptor 1) are the important genes involved in the insulin signaling pathway. MiR-128 targets all three of these proteins and decreases their expression at the mRNA and protein levels. As reported by Motohashi et al., tumor necrosis factor α (TNF-α) negatively regulates miR-128 and promotes myoblast proliferation and myotube hypertrophy by reducing the expression of miR-128 in myoblasts as well as myotubes (66, 93). In addition, bovine muscle miR-128 regulates myogenic differentiation through Sp1, which is an activator of MyoD and suppressor of CDKN1A (94). A study by Shi et al. showed that miR-128 also promotes myoblast differentiation and inhibits proliferation by targeting the coding domain sequence (CDS) region of myostatin, which inhibits myogenesis. Overexpression of miR-128 promotes myotube formation in C2C12 myoblasts and decreases proliferation (93) (**Figure 4**).

## MiR-29, miR-34c, and miR-195/497 in NF-κB-YY1 and NF-κB/IGF-1R/IR signaling

**Figure 5:**
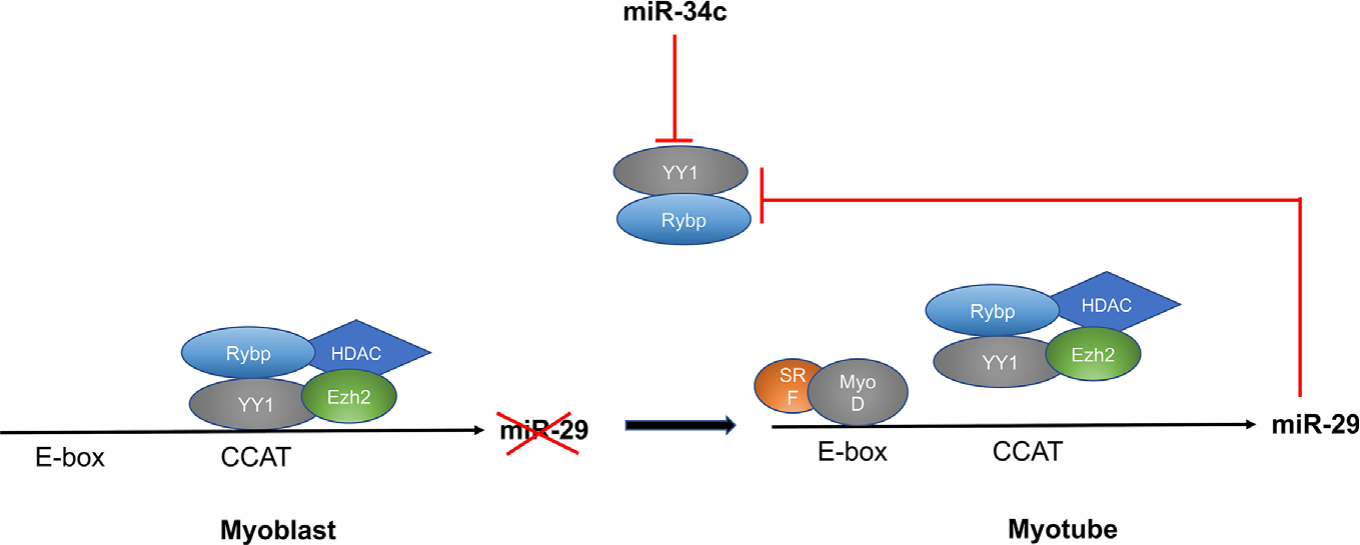
MiR-29 and miR-34c targets YY1 and regulates myoblast differentiation through the NF-κB/YY1 signaling axis.

MiR-29 regulates myoblast differentiation by directly targeting *YY1* (Yin Yang 1)—a ubiquitously expressed transcription factor that interacts with RYBP. YY1 recruits histone methyltransferase Ezh2 and histone deacetylase 1 (HDAC) upon activation by NF‐κB, which silences the expression of miR-29. At the beginning of myogenesis, decreased NF-κB signaling down-regulates YY1 and the repressive complex of YY1-Ezh2-HDAC is replaced by an activator complex containing MyoD and SRF. This results in increased expression of miR-29, which represses YY1 and RYBP expression and facilitates myogenesis as well as increasing differentiation of myoblasts into myotubes (61, 62). Another microRNA that targets YY1 and regulates myoblast proliferation and differentiation is miR-34c. Wang et al. showed that miR-34 is highly-expressed in differentiating cells and promotes myoblast differentiation as well as suppressing proliferation by directly targeting the *YY1* transcription factor (63) (**Figure 5**).

Other important non-myomiRs include members of the miR-15 family miR-195 and miR-497, which are involved in maintaining the quiescent state of satellite cells by targeting the cell cycle activators *Ccnd2*, *Cdc25*, *cyclin D1*, and *cyclin D2* (49, 50). MiR-195/497 also plays a role in regulating myoblast development. MiR-195/497 promotes myoblast proliferation by directly targeting IGF‐1R and IR genes and, during muscle growth and differentiation, the expression of miR-195 and miR-497 was significantly up-regulated. Also, expression of miR-195/497 was negatively regulated by NF-κB in C2C12 myoblasts and mouse skeletal muscle. Previous studies showed that NF-κB is constitutively increased in myoblasts and, after their differentiation, its expression is decreased (95). Taken together, these results show the role of NF-κB/IGF-1R/IR signaling in regulation of proliferation in myoblasts (50).

## Regulation of the Wnt Signaling Pathway by miR-139-5p and miR-145-5p

MiR‐139 is significantly increased during proliferation of C2C12 myoblasts and its ectopic introduction decreases their differentiation. The Wnt/β-catenin signaling pathway is an important pathway in myogenesis. MiR-139-5p binds to the 3’UTR region of *Wnt-1*, a critical regulator of Wnt/β-catenin signaling pathway. Inhibition of Wnt-1 leads to up-regulation of glycogen synthase kinase 3β (GSK-3β) and down-regulation of phospho-GSK-3β, β-catenin, and nuclear β-catenin, leading to decreased activation of downstream genes involved in myogenesis (67, 68). Another non-myomiR involved in regulation of myogenesis through the Wnt signaling pathway is miR-145-5p, which is up-regulated during C2C12 myoblast differentiation. The target of miR-145 is not yet clear but overexpression of miR-145 enhances the expression of genes involved in the endogenous Wnt signaling pathway like *Wnt5a*,* LRP5*,* Axin2*, and *β-catenin* and promotes myoblast differentiation (69).

## IGF‐1/AKT/mTOR Signaling and miR-199-3p

MiR‐199a‐3p negatively regulates differentiation of C2C12 myoblasts by directly targeting members of the IGF‐1/AKT/mTOR signaling pathway, which includes *IGF-1* and *mTOR*. This pathway plays a critical role in muscle development and IGF-1 activates both ERK1/2 and PI3K/Akt/mTOR pathways upon binding to its receptor IGF-R1. ERK1/2 regulates myoblast proliferation (96) and IGF‐1/AKT/mTOR signaling aids in protein synthesis, myotube formation, and muscle hypertrophy (97). Expression of miR-199-3p is also regulated by TGF-β, which is a well-known inhibitor of skeletal muscle differentiation (98). Overexpression and inhibition studies confirmed the role of miR-199-3p in regulating IGF‐1/AKT/mTOR signaling as overexpression of miR-199-3p decreases expression of *IGF* and *mTOR* in C2C12 myoblasts, whereas inhibition of miR-199-3p rescues IGF-1 and mTOR. Also, overexpression of miR-199-3p decreases the phosphorylation levels of AKT and mTOR, which confirms the role of IGF‐1/AKT/mTOR signaling in miR-199-3p-mediated down-regulation of differentiation in myoblasts (74).

## Expression And Function of miRNAS in the Skeletal Muscle of Other Species

Apart from the role of miRNAs in humans many miRNAs have been reported in other species including chickens, goats, and cattle—highlighting the importance of microRNAs in muscle development across species. Recently, Yin et al., explored the role of miR-9-5p in muscle development. MiR-9-5p is abundantly expressed in chicken skeletal muscles and regulates the proliferation and differentiation of myoblasts *via* the PI3K/Akt signaling pathway. This study also reports that insulin-like growth factor 2 mRNA-binding protein 3 (*IGF2-BP3*), is a direct target of miR-9-5p and IGF2-BP3 is important activator of IGF-2, which activates AKT and promotes the proliferation and differentiation of satellite cells. Overexpression of miR-9-5p inhibits satellite cell proliferation and differentiation (46).

Another microRNA, miR-16-5p, was found to be associated with skeletal muscle growth in chickens. MiR-16-5p directly targets the 3’UTR of *SESN*1, which is associated with increased proliferation and differentiation of myoblasts, along with decreased apoptosis. Also, SESN1 regulates the p53 pathway through a feedback mechanism. MiR-16-5p targets *SESN1* and, thereby, regulates the p53 pathway and promotes apoptosis, along with decreasing proliferation and differentiation of myoblasts (53).

MiR-99a-5p is also reported to regulate proliferation and differentiation of satellite cells in chickens. MiR-99a-5p targets the 3’ UTR region of myotubularin-related protein 3 (*MTMR3*) and knockdown of MTMR3 by miR-99a-5p promotes proliferation and inhibits differentiation of chicken satellite cells (99). Recently, miR-146b-3p was shown to regulate myogenesis in the chicken. *AKT1* and *MDFIC* are the direct targets of miR-146b-3p and overexpression of miR-146b-3p decreases the expression of *AKT1* and *MDFIC*; thereby, inhibiting PI3K/AKT activation, resulting in decreased proliferation and differentiation as well as increased apoptosis of myoblasts in the chicken (29).

MiRNAs have also been investigated for their role in goat skeletal muscle development. Recently, miR-125b was reported to target and inhibit the expression of insulin-like growth factor 2 (*IGF-2*) and inhibit differentiation of SMSC in this species. In the same study, the authors found that the long non-coding RNA lncR-125b directly targets miR-125b and acts as a molecular sponge for miR-125b, which decreases its expression and promotes myoblast differentiation and myotube formation (100). MiR-101a was also shown to promote goat satellite cell differentiation by targeting Ezh2 (101). In other studies, miR-487b-3p was shown to play a significant role in myogenesis in goats and overexpression of miR-487b-3p decreases C2C12 myoblast proliferation and differentiation. *IRS1* is a direct target of miR-487b-3p and it is a critical regulator of PI3K/AKT and MAPK/ERK signaling pathways. Consequently, inhibition of IRS1 expression by miR-487b-3p results in decreased PI3K/AKT and MAPK/ERK signaling and inhibition of myoblast proliferation and differentiation (102).

The role of microRNAs in skeletal muscle development in cattle is also well explored. Recently, miR-885 was shown to promote proliferation and inhibit differentiation in these animals. MiR-885 directly binds to the 3’UTR region of *MyoD1* and overexpression of miR-885 in primary bovine myoblasts upregulates proliferation and suppresses differentiation (103). Another microRNA, miR-216a, was shown to suppress myoblast differentiation and proliferation in primary bovine myoblasts by targeting *SNIP1* and *SMAD7* (104). MiR-148a-3p was also shown to regulate myoblast proliferation and apoptosis in bovine muscle cells.

*KLF6* is the direct target of miR-148a-3p and its overexpression inhibits myoblast proliferation and promotes apoptosis, while knock-down of miR-148a-3p has the opposite effect (105). MiR-107 is another microRNA reported to be highly expressed in bovine myoblasts that inhibits differentiation and apoptosis by targeting Wnt3a. In the same study, the circular RNA circFGFR4 was shown to inhibit expression of miR-107, which promotes myoblast differentiation (106). MiR-378 also plays a role in bovine skeletal muscle development as it is highly expressed in differentiating satellite cells by directly targeting the 3’UTR regions of POLA2 (52). Zhang et al., reported that miR-2400 promotes bovine skeletal muscle satellite cell proliferation by targeting myogenin (107) and miR-2425-5p also targeted *myogenin* and *RAD9A* (RAD9 homolog A) to promote proliferation and suppress differentiation (52).

In summary, miRNAs are highly conserved and exploring the role of these molecules in a variety of species may advance our understanding of miRNA-facilitated regulation in various physiological and pathophysiological processes.

## MiRNAs and Skeletal Muscle Disorders

Although the role of miRNAs in skeletal muscle cell regulation and development is well documented; a better understanding their function in muscle will provide important insights about the regulation of gene expression in health and disease. This section of the review will discuss the current understanding of miRNA regulation in skeletal muscle and their role in myopathies including sarcopenia and Duchenne muscular dystrophy (DMD). We will also discuss how miRNAs are involved in cancer cachexia, a muscle wasting syndrome associated with cancer progression.

## MiRNAs in Sarcopenia

As we age, there is gradual loss of muscle mass, function, and strength along with reduced regenerative capacity and protein synthesis. This age-associated muscle wasting is termed sarcopenia (108, 109) and miRNAs are emerging as important regulators of skeletal muscle development and homeostasis during aging.

MiRNAs can be used as biomarkers or therapeutic targets for muscle wasting as a consequence of age or disease. Several microarray-based and sequencing studies using muscle from young and old animal models and humans highlights the importance of dysregulated microRNAs in age-related muscle wasting. A study by Drummond et al. performed microarray analyses using muscles from young and old humans and found 18 differentially expressed miRNAs. In muscle samples from older individuals, eight microRNAs were significantly up-regulated (let-7a, -b, -e, and -f, as well as miR-25, miR-98, miR-195, and miR-1268) and ten microRNAs were significantly down-regulated (miR-22, miR-24, miR-27a, miR-27b, miR-30d, miR-133a, miR-133b, miR-223, miR-378, and miR-378*) when compared to young individuals. Expression of let-7b and let-7e were validated using qRT-PCR along with the expression of cell cycle regulators (*CDK6*, *CDC25A*, and *CDC34*) and *PAX7* expression. Expression of *CDK6*, *CDC25A*, *CDC34*, and *PAX7* was significantly lower in muscle biopsies from older individuals, suggesting the involvement let7 in muscle cell renewal and regeneration through the regulation of the cell cycle (110).

Another large-scale miRNA screening study investigated the role of resistance exercise on miRNA expression in skeletal muscle biopsies from young and old individuals. It was found that 26 miRNAs were differentially expressed in older muscle when compared to young. Nine miRNAs from the miR99/100 family were predicted to target the Akt-mTOR signaling pathway—highlighting the role of this family in regulating skeletal muscle mass during aging. Also, apart from miR-499a, there was no change in the expression levels of myomiRs (miR-1, miR-133a, miR-133b, miR-206, and miR-486-5p) in old individuals when compared to young individuals (111).

Rivas et al. conducted expression profiling of miRNAs in young and old men following an acute bout of resistance exercise and found that 21 miRNAs were altered in young men with no change in miRNA expression in older men. MiR-126 expression was significantly decreased in young men compared to older men after resistance exercise. Overexpression and inhibition studies in C2C12 myoblasts showed miR-126 regulates the expression of *Foxo1*, *MyoD*, *Myf5*, and *IGF-1*. Therefore, regulation of miR-126 in older patients might improve the function of skeletal muscle in aged individuals (112).

Kim et al. reported that 34 miRNAs (15 up-regulated and 19 down-regulated) were dysregulated in genome-wide miRNA profiles of gastrocnemius muscle biopsies from young and old mice. Using qRT-PCR, up-regulated miRNAs (miR-34a-5p, miR-146a-5p, miR-92b-3p, miR-155-5p, and miR-203-3p) and down-regulated miRNAs (miR-337-3p*, miR-434-3p, miR-434-5p*, miR-136-5p, and miR-148a-3p) were validated (113). In addition, miRNA profiling using a TaqMan miRNA array of quadricep muscles from young and old mice showed 57 miRNAs were differentially altered (36 down-regulated and 21 miRNAs were up-regulated.). Validation of up-regulated miRNAs (miR-7, miR-542, miR-486, and miR-686) and down-regulated miRNAs (miR-181, miR-434, miR-455, and miR-221) were also performed using qRT-PCR (114).

MiRNA profiling using next generation sequencing (NGS) of skeletal muscle tissue from young and old Rhesus monkeys was also performed. It was found that 35 miRNAs were differentially expressed in skeletal muscles of old versus young monkeys. Most of miRNAs were up-regulated, with only five miRNAs down-regulated, including miR-181a and miR-181b in old monkeys compared to young monkeys (115). In another study, miRNA profiling using NGS in myoblasts isolated from hind limb muscles of young and old mice found that miR-431 expression is significantly reduced in aged myoblasts. Overexpression of miR-431 in aged myoblasts improved regeneration, and inhibition of miR-431 also prevented myogenesis. MiR-431 directly targets the 3’UTR of *SMAD4* and effects TGF-β signaling. In aged myoblasts, SMAD4 is up-regulated, resulting in increased TGF-β signaling (an inhibitor of myogenesis), signifying miR-431 might play an important role in regulating the myogenic potential of aged myoblasts (81).

Hu et al. reported that expression of miR-29 is significantly up-regulated in the muscles of aged rodents compared to young rodents. MiR-29 targets mediators of myoblast proliferation (*e.g.* p85a, IGF-1, and B-Myb). Overexpression of miR-29 in muscle progenitor cells leads to reduced proliferation and increased senescence. Also, injection of miR-29 into the muscles of young mice resulted in an aged muscle phenotype (116). Another microRNA miR-195 was up-regulated in myoblasts isolated from aged mice compared to young mice. MiR-195 targets the anti-senescence proteins Sirtuin1 (*SIRT1*), which promotes senescence. Blockade of miR-195 significantly up-regulated the reprograming potential of old myoblasts. Blocking miR-195 in induced pluripotent stem cells derived from old myoblasts does not affect pluripotency in transformed myoblasts, providing evidence that iPSCs with expression of miR-195 blocked, have the potential to be transplanted in elderly patients (117).

Recently, a study identified the role of miR-658-5p in regulating expression of the striated muscle activator of Rho signaling (*STARS*) in young and old individuals following acute resistance exercise. MiR-658-5p expression was significantly up-regulated in older subjects as compared to young subjects. MiR-658-5p targets the 3’UTR of *STARS* mRNA and, therefore, decreases the expression of STARS in old subjects compared to young (118). MiR-143 is significantly decreased in satellite cells during aging and it is proposed to target the 3’UTR of the insulin growth factor-binding protein 5 (Igfbp5). The authors demonstrated that miR-143-3p:IGF-BP5 interactions play an vital role in age-associated changes in satellite cells (119).

## MiRNAs in Duchenne Muscular Dystrophy

Duchenne muscular dystrophy (DMD) is progressive muscle wasting X-linked recessive genetic disorder caused by out of frame mutations in the dystrophin gene resulting in severe cellular and molecular changes. MiRNAs involved in the pathological pathways activated in skeletal muscle damage and regeneration are exhibited to hasten and intensify the dystrophic phenotype (19). MiRNAs can be used as a biomarker for the early detection as well as a therapeutic target in the treatment of DMD.

MyomiRs (miR-1, miR-31, miR-133, miR-206) are also known as dystromirs as these microRNAs, which are involved in myogenesis, are also linked to muscular dystrophies. These dystromirs show potential as biomarkers as they are highly-expressed in the serum of DMD patients and are correlated with disease severity (116, 120–122). MiR-206 is a skeletal muscle-specific miRNA shown to play an important role in skeletal muscle regeneration in response to injury. Mice lacking miR-206 show hindered skeletal muscle regenerative capability following cardiotoxin injury. MiR-206 expression is significantly higher in the mdx mouse model of DMD and plays a protective role by slowing progression of DMD in these mice because miR-206 promotes satellite cell differentiation and the formation of new myofibers (123).

MiR-486 is another muscle-specific microRNA involved in myogenic differentiation. Expression of miR-486 is significantly down-regulated in DMD patients as well as in mdx mice. MiR-486 targets dedicator of cytokinesis 3 (*DOCK3*) whose expression is elevated in dystrophic muscle and regulates the PTEN/AKT signaling pathway by decreasing expression of PTEN, resulting in increased phosphorylated Akt. Overexpression of miR-486 in mdx mice improves sarcolemmal integrity and performance, enhances fiber size, and decreases nuclear centralization (124).

MiR-199a-5p is also significantly down-regulated in dystrophin-deficient zebrafish, mdx mice, and DMD human muscle biopsies. MiR-199a-5p regulates myoblast differentiation by controlling various factors involved in WNT signaling such as *FZD4*, *JAG1*, and *WNT2* (75). MiR-499 is reported as a serum biomarker in both human patients and in the Golden Retriever model of DMD (121, 125, 126). Another report by Liu et al. showed that reinstatement of miR-499 in mdx mice reduces the severity of DMD. MiR-499 targets *Fnip1*, a well-known negative regulator of AMPK, which is an activator of PGC‐1α. Inhibition of Fnip1 in myocytes activates AMPK/PGC‐1α signaling and mitochondrial function (127).

MiR-127 is a miRNA predominantly expressed in skeletal muscle and its overexpression promotes myoblast differentiation in C2C12 cells. Moreover, in transgenic mice overexpressing miR-127 there was an increase in satellite cell differentiation and enhanced muscle regeneration compared with wild-type mice. MiR-127 promotes differentiation by targeting the gene encoding sphingosine-1-phosphate receptor 3 (*S1PR3*), a G-protein-coupled receptor for sphingosine-1-phosphate. The same study showed that overexpression of miR-127 in mdx mice significantly ameliorates disease severity by promoting satellite cell differentiation (128).

Another miRNA, miR-200c, shows significantly increased expression in mdx mice. MiR-200c was reported to regulate myoblast differentiation as overexpression of miR-200c inhibits myoblast differentiation. MiR-200c was also reported to enhance ROS production and phosphorylation of p66Shc in Ser-36 by targeting *FOXO1* and *eNOS*; thereby, down-regulating skeletal muscle differentiation. Increased expression of miR-200c in mdx mice might be responsible decreased differentiation and enhanced muscle wasting and myotube loss in mdx mice (129).

Another important feature of DMD is fibrosis and collagen deposition. It is shown that decreased expression of miR-29 in mdx mice promotes fibrosis as inhibition of miR-29 in myoblasts promotes differentiation of myoblasts into myofibroblasts. MiR-29, apart from promoting myoblast differentiation *via* Akt3/NFkB/YY1 signaling, also decreases fibrosis by targeting fibrotic genes (*COL3A1*, *FBN1*, and *COL1A1*). In mdx mice, miR-29 is significantly down-regulated, along with increased muscle fibrosis and restoration of miR-29 in mdx mice by intramuscular and intravenous injection, improved the dystrophic pathology by promoting muscle regeneration and decreasing fibrosis (130). In mdx mice, muscle strength was completely restored when both mir-29 and micro-dystrophin were administered together (131).

MiR-21 is another miRNA that is significantly increased in mdx mice, and its expression is significantly correlated with increased expression of fibrotic genes such as *COL1A1* and *COL6A*. Mir-21 expression is also increased in DMD fibroblasts and further enhanced by TGF-β1 treatment. MiR-21 directly targets the 3’UTR of *PTEN* (phosphatase and tensin homolog deleted on chromosome 10) and *SPRY-1* (Sprouty homolog 1). Expression of PTEN and SPRY-1 was reduced significantly in DMD fibroblasts along with increased expression of COL1A1 and COL6A. Inhibition of miR-21 in mdx mice as well as in DMD fibroblasts reinstates expression of PTEN and SPRY-1 along with decreases in the expression of COL1A1 and COL6A; thereby, improving the disease phenotype (132). Recently, Daria et al. explored the role of synthetic preimplantation factor (sPIF) in regulating miR-21 in DMD. In DMD patient-derived myoblasts, sPIF overexpression promotes myoblast differentiation and decreases expression of COL1A1, COL1A2, and TGF-β, along with increases in the expression of the dystrophin homolog, utrophin protein. It was reported that sPIF mediated the protective effects in DMD myoblasts by upregulating lncRNA H19 and miR-675, and downregulating let-7 and miR-21 (133).

Another microRNA studied extensively in DMD is miR-31. MiR-31 maintains satellite cells quiescence by directly targeting *Myf5*. An additional target of miR-31 is dystrophin, and its expression is significantly up-regulated in mdx mice along with increased expression in human DMD biopsies compared to healthy controls. Human DMD myoblasts treated with antisense oligonucleotides to induce exon skipping showed greater dystrophin rescue upon inhibition of miR-31 (122). Also, inhibition of miR-31 using CRISPR/Cas9 editing in myotubes derived from induced pluripotent stem cells (iPSCs) of DMD patients showed restoration of dystrophin (134).

## MiRNAs in Cancer Cachexia

Another important syndrome associated with muscle wasting is cachexia. It is a multifactorial syndrome that leads to significant loss of skeletal muscle mass with or without the loss of fat mass (135–137). Cachexia is associated with the pathology of many diseases including cancer. Cancer-associated cachexia (CAX) affects 50–80% of cancer patients and is linked to 20–40% of patient mortality (138–140). Recently studies have highlighted the role of miRNAs in the pathophysiology of cancer cachexia.

A report by Mubaid et al. highlights the role of the RNA binding protein HuR in cancer cachexia. HuR plays a dual role as a promoter of muscle fiber formation and an inducer of muscle loss by interacting with the *STAT3* mRNA. Activated STAT3 promotes transcription of STAT3-dependent pro-cachectic genes, resulting in increased muscle wasting. HuR binds to the 3’UTR of the *STAT3*mRNA and prevents binding of miR-300 to the 3’UTR of STAT3, resulting in increased expression of STAT3, which leads to augmented muscle wasting (141).

MiR-203 was significantly up-regulated in colorectal cancer (CRC) patients when compared to control subjects, and miR-203 levels were correlated with the incidence of myopenia in CRC. Overexpression and inhibition studies of miR-203 in human skeletal muscle cells (SkMCs) identified *BIRC5 (Survivin)* as a target of miR‐203 in skeletal muscle cells. *BIRC5* is well known to inhibit apoptosis through inhibition of caspases 3, 7, and 9. Therefore, mir-203 promotes myopenia in CRC by inhibiting miR-203 and promoting apoptosis and muscle wasting (142).

In another study, Wei et al. showed severe cachexia in muscle cells from Lewis lung carcinoma (LLC) tumor-bearing mice compared with control mice. LLC tumor-bearing mice secrete significantly increased amounts of miR-21 through micro vesicles (MVs) and promotes apoptosis of skeletal muscle cells. MiR-21 encourages apoptosis *via* c-Jun N-terminal kinase (JNK) signaling following activation of TLR-7 receptors present on myoblasts. Moreover, there is significant increase of apoptotic muscle cell along with decreased muscle nuclei resulting in muscle wasting.

In colorectal cancer (CRC) patients, miR-21 expression was significantly increased in both tissue and serum of CRC patients compared to control subjects. Increased expression of miR-21 was negatively correlated with decreased psoas muscle mass index (PMI) of CRC patients (143). Finally, a recent study by Wouter et al. aimed to identify differentially expressed microRNAs in cachectic non-small cell lung cancer (NSCLC) patients. Using miRNA arrays, the authors reported that five miRNAs were significantly increased and 23 were significantly decreased in NSCLC patients compared to controls (144).

## Conclusions

Over the last decade, numerous studies have highlighted the importance of miRNAs as critical regulators of myogenesis along with playing a significant role in muscle-associated diseases like DMD and sarcopenia. It is quite interesting how the same transcription factors that regulate muscle-associated protein-coding genes also control the expression of muscle-specific miRNAs, suggesting interplay between miRNAs, protein-coding genes, and transcription factors. Tremendous progress has been made in recognizing muscle-specific miRNAs and their associated functional mechanisms in myogenesis. Still, there is a need to further explore the role these miRNAs in muscle-associated myopathies including DMD, sarcopenia, and cancer cachexia.

In this review, we have also discussed various molecular mechanisms associated with muscle-associated miRNAs. Attempts to unravel the function of these miRNAs not only improves our knowledge about miRNAs in muscle development and homeostasis, but may facilitate the development of miRNA-based therapies in muscle-related diseases, including DMD, sarcopenia and cancer cachexia. In the near future, microRNAs have significant potential as diagnostic, prognostic, or therapeutic tools for skeletal muscle-associated pathologies.

Despite this potential, there are substantial hurdles to address before miRNAs can be used as a therapeutic or diagnostic tool. As a therapeutics targeting miRNAs may ameliorate the symptoms of muscle-associated myopathies, the heterogeneity of miRNA expression, target specificity, and off target effects could hamper the prospects for miRNA-based therapeutics. Moreover, microRNAs may behave in a tissue-specific manner and miRNAs with opposite effects in different tissues would need to be carefully scrutinized before they can be considered for therapeutic use. Also, miRNAs often exist as a families and may contain many miRNAs with similar targets and functions. So, silencing of an individual miRNA might be rescued by other miRNAs with similar or overlapping functions. Apart from identifying targets for miRNA-based therapeutics, the development of an appropriate and stable delivery system needs to be addressed.

In conclusion, miRNAs have been established as key regulators of muscle development and function. There is ample evidence for the contribution of miRNAs in muscle development including differentiation, proliferation, and regeneration. In many skeletal muscle-associated diseases, including DMD, sarcopenia, and cancer cachexia, dysregulation of miRNAs have been reported. Altered levels of miRNAs might serve as useful diagnostic tools for early detection of myopathies. Unlike most RNAs, miRNAs are quite stable in most body fluids and abnormal expression of miRNAs in the circulation may serve as an important way to detect muscle diseases. Understanding the target genes associated with muscle miRNAs could offer useful insights into the regulation and function of muscle-related signaling pathways. MiRNAs play a significant role in skeletal muscle development and pathophysiology and may provide a means for the development of new therapeutics to treat muscle disorders.

## Author Contributions

GS: Review conception, design, manuscript writing and preparation. D-ZW: review conception, design and editing. DC: manuscript editing. All authors contributed to the article and approved the submitted version.

## Funding

Research in the D-ZW laboratory is supported by the NIH (HL125925 and HL093242).

## Conflict of Interest

The authors declare that the research was conducted in the absence of any commercial or financial relationships that could be construed as a potential conflict of interest.
